# A taxonomy for vocal learning

**DOI:** 10.1098/rstb.2018.0406

**Published:** 2019-11-18

**Authors:** Peter L. Tyack

**Affiliations:** Sea Mammal Research Unit, Scottish Oceans Institute, School of Biology, University of St Andrews, East Sands, St Andrews KY16 8LB, UK

**Keywords:** auditory–vocal feedback, compensation for noise, vocal imitation, vocal learning, vocal mimicry, complex vocal learning

## Abstract

Humans and songbirds learn to sing or speak by listening to acoustic models, forming auditory templates, and then learning to produce vocalizations that match the templates. These taxa have evolved specialized telencephalic pathways to accomplish this complex form of vocal learning, which has been reported for very few other taxa. By contrast, the acoustic structure of most animal vocalizations is produced by species-specific vocal motor programmes in the brainstem that do not require auditory feedback. However, many mammals and birds can learn to fine-tune the acoustic features of inherited vocal motor patterns based upon listening to conspecifics or noise. These limited forms of vocal learning range from rapid alteration based on real-time auditory feedback to long-term changes of vocal repertoire and they may involve different mechanisms than complex vocal learning. Limited vocal learning can involve the brainstem, mid-brain and/or telencephalic networks. Understanding complex vocal learning, which underpins human speech, requires careful analysis of which species are capable of which forms of vocal learning. Selecting multiple animal models for comparing the neural pathways that generate these different forms of learning will provide a richer view of the evolution of complex vocal learning and the neural mechanisms that make it possible.

This article is part of the theme issue ‘What can animal communication teach us about human language?’

## 1. Introduction

When an animal vocalizes, it must generate the right pressure in its lungs, adjust the tension and vibration rate of its vocal cords and configure the upper respiratory tract to produce the sound. All of these actions must be coordinated with plans for respiration and swallowing. Research with vertebrates from fishes to mammals has shown that much of the complex coordination of the motor nuclei involved in these components occurs in the brainstem (teleost fishes: [1]; non-human primates: [2]). Bass *et al*. [3] have argued that the vocal pattern generators of fishes and all tetrapod vertebrates evolved from an ancestrally shared developmental compartment of the brainstem. Stimulation of the appropriate areas of the brainstem can generate complete vocalizations, suggesting that central pattern generators in the brainstem encode all the information required to integrate all of these respiratory, phonatory and articulatory movements to produce a sound.

Some vertebrate species have evolved neural mechanisms that allow them to go beyond fixed motor programmes for vocalization and to produce sounds that match a wide variety of sounds that they hear. These mechanisms for vocal learning are critical for human speech and music. Some other animals also have this capacity for vocal learning [4], which enables comparative studies on the evolution, development and neural basis of human speech. Extensive research in humans and many songbird species has shown they learn to speak or sing by listening to acoustic models, forming auditory templates and then learning to produce vocalizations that match the template [5]. This ability for vocal learning is defined by Janik & Slater ([4], p. 59) as learning ‘where the vocalizations themselves are modified in form as a result of experience with those of other individuals’. Janik & Slater [6] distinguish vocal production learning, which involves changing the acoustic parameters of a vocalization, from contextual learning, which involves associating or producing an existing signal in a new context. Here, I am only concerned with vocal production learning, so will use ‘vocal learning’ as synonymous with ‘vocal production learning’.

In this paper, I point out that there are several limited forms of vocal learning that qualify by the Janik & Slater [4] definition as vocal production learning, but which may involve fine-tuning an inherited motor pattern rather than matching a learned template. Janik & Slater ([6], p. 8) mention ‘the possibility of learned gradual parameter changes within call types' as a form of vocal learning that has received little attention. These limited forms of vocal learning may involve neural networks that differ from those required for complex vocal learning, which I define by the need to hear a sound to form a learned auditory template before the animal can develop a vocalization that matches the template.

Janik & Slater [4] argue that vocal learning appears to have a very limited distribution among birds and mammals. They argue that the strongest evidence for vocal learning stems from experiments that test whether an animal can learn to copy sounds of another species or to copy artificial sounds. This certainly qualifies as complex vocal learning by my definition. Vocal learning is more commonly involved in the development of a species-specific repertoire, but some species do not restrict the learning of auditory templates to species-specific sounds. Hindmarsh [7] suggests that about 20% of passerine birds mimic the sounds of other species. Humans have long trained birds such as parrots and songbirds to imitate speech, and hummingbirds can learn to copy the aberrant song of a cross-species hybrid [8] or replace their song as they hear new song types [9], but this kind of vocal learning has not been demonstrated for any of the 20 or so other orders of birds. The three avian orders with evidence for vocal learning are only distantly related, suggesting that vocal learning evolved independently three times in birds [10]. Evidence for complex vocal learning in mammals also has a spotty taxonomic distribution, with just a few cases of non-human mammals showing the ability to copy novel sounds. For example, a harbour seal (*Phoca vitulina*), who was raised in a Maine home, spoke English with a New England accent [11]. An Indian elephant (*Elephas maximus* indicus), who was raised in a zoo, learned to produce the Korean words used by his trainer as commands [12]. It is more difficult for humans to raise obligate aquatic mammals such as dolphins in close proximity, but bottlenose dolphins (*Tursiops truncatus*) can be trained to imitate computer-generated patterns of frequency modulation [13]. Seals and elephants appear to represent two independent evolutionary origins of vocal learning with a laryngeal sound production mechanism, and toothed whales, which have evolved a novel sound production organ [14], represent a third origin of these vocal learning capabilities among non-human mammals.

Given the importance of vocal learning for humans, there is a surprising lack of evidence for vocal learning among non-human primates. Intensive efforts to train apes to speak met with failure [15]. Experiments that attempted to disrupt development of vocalizations in squirrel monkeys (*Saimiri sciureus*) showed that infants which were deaf or had mute parents still could produce normal vocalizations [16,17], suggesting that the vocalizations developed from central pattern generators that do not require auditory input from conspecifics. And evidence from cross-breeding strains of primates that have different vocalizations suggests that some variations in acoustic structure are inherited [18,19]. Vocalizations with acoustic structures that are inherited and whose development does not require auditory input have been called innate vocalizations [2].

Over a century of neurological research has demonstrated that the ability of humans to speak depends upon cortical networks for which there is little evidence in non-human primates. Stimulation or lesions of specific areas of cerebral cortex can produce or disrupt speech in humans, but stimulation or lesions of homologous areas in non-human primates do not affect their vocalizations [20,21].

Given the lack of vocal learning in non-human primates, songbirds have been the dominant animal model for studying the neural mechanisms of vocal learning over the past few decades. The forebrain of birds is organized into nuclei, a structure which differs from the multi-layered cortex in mammals, but in spite of this significant difference, there are striking parallels in the organization of neural networks for vocal learning in songbirds and humans. Jarvis [22] argues that songbirds and humans each have two pathways in the forebrain for vocal learning, an anterior pathway that is required for learning the acoustic structure and sequencing of sounds and a posterior pathway responsible for production of learned sounds. Similar to the contrast between humans and non-human primates, the nuclei specialized for song learning in oscine songbirds are not present in suboscine birds which develop normal songs in the absence of exposure to songs of their species [23].

In addition to learned vocalizations such as music and language, humans also have innate vocalizations such as crying and laughter [24], and songbirds with learned songs also produce calls, most of which are thought to be innate [25]. For many decades, neurologists have located brain lesions that affect the human voice by assuming that it is activated by two separate mechanisms; the classic description suggests that one generates innate vocalizations for emotional expression similar to those of other mammals and that another generates speech under volitional control using cortical networks that evolved de novo in our human ancestors [26]. Studies of learned and innate vocalizations have led to the conclusion that there are two separate pathways controlling vocalization in mammals [2] and songbirds [27], one controlled by innate pattern generators in the reticular formation of the brain stem and another parallel pathway that evolved later and is controlled by telencephalic processes that generate patterns for learned vocalizations [28].

In mammals, the vocal pattern generators in the brain stem are activated by centres in the periaqueductal gray (PAG) in the mid-brain, which are responsible for initiating a vocalization and controlling its intensity, but which do not appear to control its patterning [2]. The PAG itself can generate unplanned responses such as a pain cry to a painful stimulus, but voluntary initiation of an innate vocalization requires the anterior cingulate cortex (ACC), which projects to the PAG [28]. All of the muscles involved in sound production that are activated by the brainstem vocal pattern generators are also represented in the motor cortex. Jürgens [2] argues that primates have a parallel pathway with direct connections from laryngeal motor cortex to the reticular formation areas that project to the motor nuclei involved in vocalization, bypassing the PAG. Based on observations of Kuypers [29] that humans have strong projections from the motor cortex directly to the motor nuclei involved in vocalization, but that cats and non-human primates do not, Fitch *et al*. [30] specify a Kuypers/Jürgens hypothesis that direct connections from the motor cortex to the primary motor neurons controlling the vocal apparatus are required for complex vocal learning.

The innate and learned pathways for vocalization may be separate, but they cannot operate independent of one another. Doupe & Kuhl ([31], p. 599) argue that ‘both songbirds and humans have high-level forebrain areas that control the pre-existing hierarchical pathways for vocal motor control’. Simpson & Vicario ([32], p. 1541) ‘suggest that the learned features of oscine songbird vocalizations are controlled by a telencephalic pathway that acts in concert with other pathways responsible for simpler, unlearned vocalizations’. They studied the long call of zebra finches (*Taeniopygia guttata*). Females develop this call without learning, but males use the same telencephalic pathways involved in song learning to learn features of the call. When the learning pathways are blocked in males, males revert to a call similar to the innate female call, suggesting that the learning pathways suppress the innate pattern without modifying the innate motor programme. Simonyan & Horwitz [28] argue that voice control in humans requires coordinating the interactions between the pathways for learned and innate vocalizations, and they argue for mechanisms in the ACC and also in the brain stem.

## 2. Limited vocal learning has a broader taxonomic spread than complex vocal learning

Researchers interested in animal models for complex vocal learning that is supported by cortical networks as described above must be able to differentiate complex vocal learning from limited vocal learning, which may have different functions and be generated by different neural networks. Petkov & Jarvis [33] argue for a spectrum of complexity in vocal learning, and they assume that the more complex the learning, the fewer species will have the ability. They are agnostic as to whether the actual distributions of vocal learning ability are smooth and continuous or whether there are step functions with different classes of animals having different categories of vocal learning. Here, I focus on distinguishing different categories of vocal learning that may involve different neural pathways. I define *limited vocal learning* as the ability to fine-tune acoustic features of species-specific vocalizations that can develop in the absence of auditory input because innate motor programmes can generate the species-specific pattern. This stands in contrast with *complex vocal learning* which is defined by the need to hear a sound to form a learned auditory template before the animal can develop a vocalization that matches the template. The vocal learning literature tends to emphasize that complex vocal learning has a sparse and patchy taxonomic distribution, but here I argue that limited vocal learning can have a much broader taxonomic distribution.

As bioacousticians have developed better abilities to quantify subtle differences in acoustic features, evidence has accumulated that hearing the sounds of other individuals can modify the acoustic structure of vocalizations often thought of as innate. An important experimental design for this phenomenon involves measuring acoustic features of vocalizations of animals before and after they are housed together. For example, Nowicki [34] showed that the calls of a group of black-capped chickadees (*Parus atricapillus*) converged on the central tendency of features within the group within a week of when it was housed together. Bird calls have traditionally been thought of as innate [25], but the Nowicki [34] evidence for convergence demonstrates vocal learning by the definition of Janik & Slater [4]. Hughes *et al*. [35] showed that chickadees raised in isolation develop some notes in their call with acoustic features within the normal range of wild birds. This suggests a central pattern generator that can develop the note in the absence of hearing other conspecifics producing it. However, other call notes are more similar to wild-type in chickadees that experience the calls of others, demonstrating a role for limited learning in parts of the same call (see ([36], pp. 25–33) for further discussion of innate and learned factors in vocal development).

Vocal convergence has been reported for many species whose vocalizations are thought to be produced by central pattern generators in the brainstem. In many non-human primate species, the calls of individuals become more similar when they live together (pygmy marmosets *Cebuella pygmaea*, [37]; cotton-top tamarins *Saguinus oedipus*, [38,39]; and chimpanzees *Pan troglodytes*, [40–43]). Even the contact calls of young goats (*Capra hircus*) converge when they are housed together [44]. Vocal convergence has even been demonstrated in playback studies of white-lipped frogs, *Leptodactylus albilabris*, where 12 of 17 frogs exposed to conspecific sounds converged on the dominant frequency of the calls [45]. This broad taxonomic distribution of convergence for vocalizations thought to be innate suggests the need to distinguish this more limited form of vocal learning from the complex form produced by songbirds and humans using specialized neural pathways in the telencephalon [22,46].

A key reason to distinguish limited from complex vocal learning is the hypothesis that limited vocal learning may not require cortical networks used to form and match auditory templates and may be achieved using other neural pathways. Comparative analysis of which neural pathways have been recruited for which tasks can help us to understand how different parts of the central nervous system (CNS) solve different vocal communication problems. A more careful analysis that distinguishes different vocal learning capabilities in the animal kingdom will also allow us to make more educated selections of species for studying the evolution of neural mechanisms that enabled human language and music.

## 3. Auditory–vocal feedback and limited vocal learning need not involve cortical networks

There is abundant evidence for a variety of ways in addition to vocal learning that auditory input affects vocal behaviour. I use the term auditory–vocal feedback (AVF) to include both vocal learning and also changes in vocal behaviour owing to auditory input that does not involve experiencing other individuals. The study of complex vocal learning has focused on specialized neural circuits in the telencephalon, but there are many other sites where auditory feedback has been shown to influence vocalization. Bass & McKibben [1] argue that fishes, birds and mammals all have centres at forebrain, mid-brain and hindbrain levels that integrate the auditory and vocal systems, providing multiple sites where AVF can take place. Labelling studies in sound-producing fish species have uncovered vocal-acoustic complexes in the hindbrain, mid-brain and forebrain that receive input from the auditory system and produce output that generates vocalizations.

Several different functions have been identified for modulation of vocal output by auditory input. Compensation for noise is one of the most ubiquitous forms of AVF because all animals that communicate acoustically face the problem of making their signal detectable in varying levels of ambient noise [47]. A variety of mechanisms can be used to compensate for noise, including making the signal louder, increasing the length or redundancy of the signal, or shifting the signal outside of the frequency band of noise [48]. Birds and mammals have been shown to use all of these mechanisms. The ability to call louder in elevated noise is called the Lombard effect after the author who first described it in humans [49]. The Lombard effect has since been found in all birds and mammals tested [50], but it is not limited to birds and mammals. Even a frog species has been shown to call more loudly after louder playback of frog calls [45]. There is evidence that some anurans [51,52] and even an insect (bow-winged grasshoppers, *Chorthippus biguttulus*; [53]) can shift the frequency of their calls upwards when in the presence of low-frequency noise. These results emphasize the taxonomic breadth of mechanisms to compensate for noise. Brumm & Zollinger [50] suggest that the Lombard effect has a very old history in birds and mammals, and they argue that either it independently evolved in both taxa or originated in a common ancestor.

The Lombard effect appears to be influenced by auditory–vocal (AV) interactions at all levels of the brain. Nonaka *et al*. [54] surgically separated the brainstem from the cerebrum in cats to show that the brainstem alone is sufficient to elicit the Lombard effect. The brainstem of the squirrel monkey contains AV neurons that respond when the monkey hears noise and also when it produces its own vocalization, leading Hage *et al*. [55] to suggest that the brainstem may mediate the Lombard effect in this species as well. The Lombard effect was initially viewed as a reflex, but noise compensation is now viewed as more complex, involving pathways that involve the mid-brain and cortex in primates. In non-human primates, the PAG not only serves a gating function for vocalization, but also controls the acoustic intensity of a vocalization [2]. Some AV neurons in the PAG respond more strongly when a squirrel monkey hears conspecific vocalizations while it is also vocalizing, suggesting that mid-brain circuits could also generate the Lombard effect. Eliades & Wang [56] demonstrated that when marmoset monkeys (*Callithrix jacchus*) vocalize in noise, neurons are activated in the auditory cortex whose activity predicts the extent of later Lombard compensation, suggesting cortical involvement in some neural networks that produce the Lombard effect, at least in primates.

Several sophisticated forms of AVF have evolved in echolocating bats. When an echolocating bat encounters a conspecific that is vocalizing at the same frequency, it may shift the frequency of its signals in what is called a ‘jamming avoidance response’. Some bats also shift the frequency of their outgoing echolocation signals so that the Doppler-shifted returning echo occurs at a favoured frequency. There is some evidence that the neural networks for this Doppler compensation involve processing in the mid-brain. Metzner [57] describes AV neurons in the mid-brain of the rufous horseshoe bat (*Rhinolophus rouxi*) that respond both to vocalizations of the bat and to hearing simulated echoes. He then develops a model to explain how the observed AV neuron behaviour can produce the observed Doppler compensation. Humans have also been shown to shift their vocalization frequencies if their auditory feedback is artificially frequency shifted [58].

## 4. Do vocal feedback mechanisms that operate on timescales of seconds differ from vocal learning during weeks or more of development?

The jamming avoidance response meets the Janik & Slater [4, p. 59] definition of vocal learning as learning ‘where the vocalizations themselves are modified in form as a result of experience with those of other individuals’. However, it involves a more rapid feedback response than classic vocal learning in which an auditory template is formed during exposure to a sound after which the ability to produce the previously heard sound is gradually learned from repeated attempts to match vocal output to the template. The task of shifting one feature, such as frequency, based on auditory input heard at the same time is likely to select for different neural networks than those that support learning a suite of features over a longer period of vocal development. Once song or speech has stabilized in its adult version, auditory feedback is still used for error correction on timescales of a second or so. This real-time feedback may involve neural networks with different components than those required for vocal development. The need for rapid processing may select for transmission over fewer synapses at lower levels of the CNS closer to the primary auditory inputs and vocal motor outputs. Conversely, pathways that include the cortex may be better structured for slow formation of a memory of a flexible auditory template during repeated experience of a model sound, for matching to a variety of potential acoustic features and for vocal development that is affected by more general learning processes as well.

Humans and songbirds often separate the timing of the formation of the auditory template from the process of comparing vocal production to the template, assessing any mismatch and correcting the error. This process of learning to produce a vocalization through trial and error correction can take a long time. After formation of the auditory template, young humans and songbirds first produce vocalizations that are far from the adult version: subsong in songbirds and babbling in young infants. Fitch [59] suggests that a babbling phase may be a necessary component of complex vocal learning. This hypothesis can be tested by studying vocal development in other species capable of vocal learning. Evidence from bottlenosed dolphins (*T. truncatus*) supports the hypothesis. Infant bottlenose dolphins in captive settings first produce a variable repertoire of unstereotyped whistles but develop individually distinctive signature whistles by 1.5–2.5 months of age [60].

However, babbling may not necessarily represent learning to match vocal output to a learned auditory template. Knörnschild *et al*. [61] report that pups of the greater sac-winged bat, *Saccopteryx bilineata*, combine elements of all adult vocalizations into unstructured bouts that they describe as ‘babbling’. Knörnschild *et al*. [62] show that as young greater sac-winged bats develop from 2 to 10 weeks of age, they modify precursor songs to match the song of the adult male in their group, whether that male is their father or not. Knörnschild *et al*. [62] describe this as complex vocal imitation. I view this as clear evidence for vocal learning, but not for complex vocal learning by my definition. The key missing evidence is whether the elements of adult vocalization produced by pups at two weeks of age are produced by innate vocal motor programmes which are then fine-tuned by limited vocal learning, or whether the two-week-old pups are already forming an auditory template and the ‘babbling’ represents attempts to match an unstructured series of templates. Only the latter case would represent complex vocal learning by my definition. Comparison with humans and songbirds suggests that this latter alternative would involve unusually rapid learning of the template and efforts to produce vocalizations to match it.

The overproduction of a high diversity of vocalizations in the young followed by a narrowing of the vocal repertoire need not always indicate vocal learning. In species where the young produce a large and variable vocal repertoire, social interactions may reinforce selection of some sounds for the adult repertoire [63]. This reinforcement can influence vocal development whether or not it involves template matching. Takahashi *et al*. [64] suggest that marmoset (*Callithrix jacchus*) parents may direct the transition in their infants from immature to adult calls by calling in response to particular infant calls. This suggests a role for this kind of reinforcement in selecting the mature vocal repertoire, even for some species that may not have complex vocal learning.

## 5. Sequence learning to develop diverse and complex displays

An important consequence of complex vocal learning in human speech and birdsong is that it can generate a huge diversity of utterances, many more than can probably be generated by independent auditory templates or innate vocal motor programmes. Humans and songbirds construct such a large number of utterances by segmenting them into subunits and memorizing the serial order of subunits. A population of neurons in an upper vocal control centre in the zebra finch appears to have a unique pattern of firing at each precise time in the overall song, providing sequencing information to the lower vocal centres to generate the timing for a sequence of the subunits [65]. When songbirds listen to song, they also appear to process groups of notes together [66], suggesting a hierarchy of perceptual processing. There is some evidence that non-human mammals with complex vocal learning also may use subunits to generate and categorize a diverse vocal repertoire using processes similar to those studied in humans and songbirds. Pace *et al*. [67] analysed humpback song using short subunits, which produced a more accurate classification than using whole syllables as the basic unit of analysis. Some toothed whale species also develop diverse repertoires of complex calls that appear to be made up of subunits (killer whales: [68,69]; bottlenose dolphin whistles: [70]).

Human speech is typically analysed in a hierarchy of phoneme, word and sentence, and birdsong is traditionally analysed in terms of a hierarchy of notes, syllables and motifs that make up a song. The distinction between bird calls and song is that calls are ‘short discrete vocalizations uttered irregularly or in isolation’ while songs ‘are longer, more complex stereotyped call sequences that are repeated frequently’ ([71], pp. 536–538). Some of the best examples of vocal learning in animals come from songs, but animals can construct complex songs from a repertoire of innate syllables. For example, Holy & Guo [72] discovered that male mice sing complex songs made up multiple syllable types emitted in repeated sequences. However, Portfors & Perkel [73] review several studies testing for vocal learning in mice and they conclude that mice are not capable of vocal learning. This suggests that mice, like some birds, learn to construct complex songs from learning sequences of innate syllables.

Understanding the potential for sequence learning provides a different perspective on vocal learning. Animals with complex vocal learning must hear vocalizations to learn them, but the templates may occur at the subunit level. Similarity in the syllables that make up the learned songs across populations of a songbird species has led Marler [74] to suggest that songbirds have innate predispositions to learn templates for specific elements of conspecific songs. For example, the multitude of swamp sparrow songs can be described in terms of six note types and the distinctive songs of each population are made up of different sequences of these notes. These results emphasize the importance of determining the basic units of vocalizations that are learned by template matching and to differentiate them from series of these units that can be learned through sequence learning.

## 6. Evidence for complex vocal learning in non-human mammals

I have argued here that limited vocal learning, which has a broader taxonomic distribution among mammals than complex vocal learning, may not provide good animal models for studying complex vocal learning because limited vocal learning may involve fine-tuning of brainstem vocal pattern generators and need not involve specialized telencephalic networks. This suggests the importance of critically evaluating evidence for complex vocal learning among potential study species. Following Janik & Slater [4], the strongest evidence for complex vocal learning is taken here as the ability to copy sounds that are not part of the normal species-specific repertoire. The most striking cases of this ability occur when an animal can learn to imitate human speech well enough for native human speakers to understand the words. Here, there is no chance that the animal is simply matching a vocalization it hears with the closest one in its species-specific repertoire. Cases where individual animals copy complex vocalizations that are not shared as a species-specific repertoire but are individual-specific or population-specific may provide adequate but weaker evidence for complex vocal learning in which the subject must learn a new acoustic template for the vocalization and then learn to develop a vocalization that matches the template.

Even though birds have a different sound production organ from mammals, humans have learned how to train several avian taxa including some parrots and mynah birds to imitate human speech (parrots: [75]; Mynah birds: [76]). By contrast, there are only a few cases where mammals raised in captivity have developed intelligible speech sounds. Recent modelling of the vocal tracts of monkeys has shown that monkeys would be capable of producing sounds like those of human speech if they had the neural capacity for vocal learning [77,78], but there is only weak evidence for such imitation. A male harbour seal that was raised by humans since he was born started to produce about eight different English phrases as he reached sexual maturity [11]. He became highly vocal for several years before refining his production of speech sounds, and he had to adopt an unusual posture to produce them. This imitative ability is not limited to one seal. Stansbury & Janik [79] trained grey seals to match sequences of musical notes or to match formant frequencies of human vowel sounds, using a careful design to make sure that acoustic features of the copies did not appear in the pre-exposure repertoire of the subjects and were not part of the normal grey seal repertoire in the wild. Reichmuth & Casey [80] also review other evidence for vocal learning in seals, sea lions and walruses. Stoeger *et al*. [12] describe a case of a male Asian elephant (*E. maximus*) that was able to imitate Korean words with enough precision for native speakers to understand the words. In order to imitate speech, the elephant stuffed his trunk in his mouth to render the acoustic properties of his upper vocal tract more like that of humans. These imitated speech sounds had acoustic features that mapped well onto human speech but were very different from those of normal seal or elephant vocalizations. These cases provide very strong evidence that the animals needed to learn new acoustic templates and use trial and error learning to produce vocalizations that matched them.

### (a) Cetaceans

Lilly [81] reports that a bottlenose dolphin was able to match the number and duration of human speech sounds and there are three reports of beluga whales imitating human speech [82–84], but none provide cases of imitation of words as convincing as those shown for skilled avian mimics or the harbour seal and Asian elephant discussed above. The first paper on beluga vocalizations recorded in the wild stated that ‘Occasionally the calls would suggest a crowd of children shouting in the distance’ [85, p. 143], which highlights the importance of making sure that sounds interpreted as ‘copies’ are not present in the normal pre-exposure repertoire of the species. This is necessary to rule out the possibility that the subject was just matching speech with the closest pre-existing call in its repertoire rather than actually copying a speech sound. Janik [86] provides a general review of vocal learning in cetaceans. The best evidence for complex vocal learning in cetaceans involves bottlenosed dolphins copying synthetic frequency modulated tones, which were similar in general acoustic structure to dolphin whistles, but which had contour patterns that were not present in the pre-exposure repertoire [13]. Several other papers claim to find evidence for vocal learning in toothed whales. Favaro *et al*. [87] report that a Risso's dolphin (*Grampus griseus*) cross-fostered with bottlenose dolphins produced whistles more like a dolphin in its pool than like wild *Grampus*, but similarity of whistles from different delphinid species [88] makes this kind of cross-fostering experiment less robust than for species with less overlap in vocal repertoires. Abramson *et al*. [89] trained a captive killer whale to match sounds either from her own calf or from a human but did not use the same methods to test for matches in the pre- and post-exposure repertoires, which hinders interpretation. Few studies of vocal learning in toothed whales meet the gold standard of quantifying the pre-exposure repertoire of the subject, designing signals that clearly differ from this repertoire and demonstrating accurate matching in the exposure or post-exposure repertoires as well as Richards *et al*. [13] study. For animals that can be held in a managed setting, experiments that train subjects for imitation of carefully constructed stimuli such as those of Richards *et al*. [13] and Stansbury & Janik [79] represent an important method for testing for complex vocal learning by imitation.

The strongest evidence for complex vocal learning in baleen whales stems from the process by which individual humpback whales copy changes in the song of their populations. Within a population, the song changes over time [90], with each individual whale tracking the changes of the population [91]. Noad *et al*. [92] report that when a few males from the humpback population on the west coast of Australia brought their song to the east coast of Australia, their song was picked up by the entire east coast population within 2 years. These examples of copying and tracking changes within and between populations demonstrate that whales must learn the acoustic structure of each unit of the song as well as the sequence of units that make up the song. Bowhead whales (*Balaena mysticetus*) produce such a diverse set of songs with so much interannual variability [93] as to also provide evidence for complex vocal learning.

### (b) Bats

Evidence for vocal learning in bats is described in detail by Vernes & Wilkinson [94]. They report no evidence for bats copying sounds of other species or novel synthetic sounds. I consider most cases of vocal learning reported in bats to reflect limited vocal learning (see table 1 of Vernes & Wilkinson [94]) as they involve vocal convergence (e.g. [62,95–98]) or differences in vocal development of isolated bats versus those exposed to sound playback or conspecifics (e.g. [99,100]), which could involve convergence for the exposed animals. As Vernes & Wilkinson [94] describe, bats are more accessible for neurobiological research than many of the other mammals shown to have vocal learning skills. This makes them attractive for testing for differences in the neural underpinnings of AVF and limited versus complex vocal learning. Lattenkamp & Vernes [101] report that bats are subjects of only about 2% of studies published on vocal learning, and no studies have tested imitation of novel sounds in bats. This emphasizes the importance of systematically studying which taxa are capable of which forms of vocal learning before reaching final conclusions about the presence or absence of these skills.

## 7. Conclusion and future directions

I have defined a classification system for different forms of AVF and vocal learning that evolved to solve different problems and that are likely to involve distinct mechanisms. As Vernes & Wilkinson [94] argue, studying the evolution and neural underpinnings of vocal learning demands distinguishing between these different forms. I define complex vocal learning by the need to hear a sound to form a learned auditory template before the animal can develop a vocalization that matches the template. I contrast this with limited vocal learning defined as the ability to fine-tune acoustic features of species-specific vocalizations that can develop in the absence of auditory input because innate motor programmes can generate the species-specific pattern. Complex vocal learning has been associated with specialized telencephalic networks in humans and songbirds and has been described for a much narrower set of species than has limited vocal learning. Testing whether these telencephalic networks are required for complex vocal learning but not for limited vocal learning requires careful selection of which species are appropriate for representing each form of learning.

The taxonomic distribution of complex vocal learning suggests several independent origins in birds and mammals [102]. However, the discovery of vocal learning in species such as elephants and seals has depended upon fortuitous cases of individuals being discovered to have learned to copy human speech; it is probably present but undiscovered in other species. The strongest evidence for complex vocal learning stems from the ability to copy sounds that differ from the normal conspecific repertoire. However, animals may use complex vocal learning to form auditory templates of their normal species-specific vocalizations and then to match them. Some species have evolved more selective predispositions to limit learning of auditory templates to species-specific vocalizations, while others may imitate sounds that are not typical of their species. We are more likely to detect complex vocal learning in species with less stringent predispositions, but testing for complex vocal learning must include species that only form templates for their normal species-specific vocalizations. The critical point for distinguishing complex from limited vocal learning is whether subjects require auditory input to develop their normal species-specific vocalizations, or whether a central motor programme allows these to develop in the absence of auditory input. Some of the procedures used in the past for testing this point, such as deafening subjects before they have a chance to hear conspecifics, are unlikely to meet modern standards for welfare of many of the taxa discussed here. Higher welfare standards should stimulate alternative approaches.

There is relatively strong evidence for innate calls in non-human primates, which have only been shown to have limited capacity for vocal learning. However, tests for vocal learning are so limited for birds and mammals that we cannot establish the presence or absence of specific vocal learning capacities in most families. Tests for the presence of specialized telencephalic networks for vocal learning are similarly limited in different families of bird and mammal. A broad comparative study of the origins of vocal learning demands a systematic selection of species with respect to mammalian and avian phylogeny [33]. Strategic selection of species for testing the absence of vocal learning in critical parts of the phylogeny is just as important as identifying taxa with different forms of vocal learning. Only with such efforts can we develop confidence about the phylogenetic positions of independent origins (or losses) of vocal learning, and of the evolutionary relationships between different forms of AVF and vocal learning.

The quest to understand which neural networks are involved in which forms of vocal learning, and how they perform the necessary information processing will also require careful selection of different model species [30,101]. In this paper, I have explored a series of questions about neural pathways for the different forms of vocal learning. Taking the broadest perspective on AVF: where are centres in the brain where auditory input converges on networks that generate vocal motor output? What are their functions and how conserved are they across the vertebrates? How do pathways for AVF and vocal learning interact with pathways for innate vocalizations? Important questions about limited vocal learning include: what and where are the neural networks that fine-tune vocal motor programmes based on auditory input? How different are the demands of real-time feedback mechanisms versus slow vocal learning during ontogeny, and how might these differences select for different neural pathways? There are long-standing questions about complex vocal learning: does complex vocal learning require telencephalic networks in all species with the trait? If so, what characteristics favour the telencephalon for complex vocal learning? How homologous are the independently evolved telencephalic networks? Do all of these independently evolved telencephalic networks have direct connections to motoneurons that innervate the vocal musculature? How does the template form? How does the CNS estimate an error signal between vocal output and template? How does the CNS modify vocal output to correct the error? How does sequence learning interact with template matching? How specialized are the neural pathways by which social reinforcement affects vocal development? How different are the pathways by which animals learn to develop new signals through ontogeny versus correct errors in production of mature vocalizations in real time?

Testing hypotheses about neural pathways required for the different forms of vocal learning requires careful selection of study species. Methods may be available to test some of these hypotheses in the full range of species for which complex vocal learning is thought to be present or absent. For example, neuroanatomical studies should be able to test for specialized telencephalic nuclei and pathways over a broad range of avian taxa for which freshly preserved specimens are available. Current methods for testing the Kuypers/Jürgens hypothesis that complex vocal learning requires direct connections between laryngeal motor cortex and motoneurons that innervate vocal musculature require invasive axonal tracing procedures with living animals. These procedures may routinely be used for some model species in neurobiology, but they are unlikely to meet modern welfare standards for many other species. However, testing whether complex vocal learning correlates with more robust tracts between motor cortex and the brainstem nuclei that innervate vocal musculature (nucleus ambiguus for the larynx and facial motor nucleus for toothed whales, [103]) may be possible using post-mortem tract tracing even with species such as elephants [104] and marine mammals (e.g. [103]). I have suggested that more attention needs to be paid to the role that auditory input may play with mid-brain and lower brainstem vocal pattern generators. Invasive neurobiological methods may be able to test these ideas with some species that are model systems in neurobiology, including species that are not capable of complex vocal learning. The hypothesis that limited vocal learning may involve mid-brain and lower brainstem vocal pattern generators and that complex vocal learning requires cortical networks may be able to be tested at a coarse scale using non-invasive or minimally invasive neurobiological methods as suggested by Ravignani *et al*. [105]. The success of non-invasive methods in studying neural mechanisms underlying human language should challenge those interested in vocal learning to develop ways to apply these methods to a broad enough taxonomic range of subjects for a comparative analysis of vocal learning and AVF.

It is important not to close without considering the ethics of working with the broad array of species discussed here. Many of the species that are capable of vocal learning are endangered, threatened or protected, and it is critical that access to subjects have no negative impact on wild populations. Research on such species should be designed to improve their conservation status, potentially by enhancing our appreciation of their capabilities. Animal welfare must be carefully taken into account as part of the process of selecting methods and species as subjects for this research. As with work with human subjects, the development of methods to study the neural processes involved in vocal learning must incorporate stringent standards for the welfare of the subjects. The last few decades of development of neurobiological methods that are appropriate for human subjects should encourage development of similarly appropriate methods for animal studies. The goal for selecting some of these species as models for understanding vocal learning should not just be based upon lower welfare standards compared to humans, but rather for their power in terms of comparative studies of the evolution of neural mechanisms that underpin the different forms of vocal learning described here.

## Data accessibility

This article has no additional data.

## References

[1] Bass AH, McKibben JR 2003 Neural mechanisms and behaviors for acoustic communication in teleost fish. Prog. Neurobiol. 69, 1–26. (10.1016/S0301-0082(03)00004-2) 12637170

[2] Jürgens U 2009 The neural control of vocalization in mammals: a review. J. Voice 23, 1–10. (10.1016/j.jvoice.2007.07.005) 18207362

[3] Bass AH, Gilland EH, Baker R 2008 Evolutionary origins for social vocalization in a vertebrate hindbrain–spinal compartment. Science 321, 417–421. (10.1126/science.1157632) 18635807 PMC2582147

[4] Janik VM, Slater PJB 1997 Vocal learning in mammals. Adv. Study Behav. 26, 59–99. (10.1016/S0065-3454(08)60377-0)

[5] Konishi M 2010 From central pattern generator to sensory template in the evolution of birdsong. Brain Lang. 115, 18–20. (10.1016/j.bandl.2010.05.001) 20955898

[6] Janik VM, Slater PJB 2000 The different roles of social learning in vocal communication. Anim. Behav. 60, 1–11. (10.1006/anbe.2000.1410) 10924198

[7] Hindmarsh AM 1984 Vocal mimicry in starlings. Behaviour 90, 302–324. (10.1163/156853984X00182)

[8] Wells S, Baptista LF 1979 Displays and morphology of an Anna×Allen hummingbird hybrid. Wilson Bull. 1, 524–532.

[9] Araya-Salas M, Wright T 2013 Open-ended song learning in a hummingbird. Biol. Lett. 9, 20130625 (10.1098/rsbl.2013.0625) 24068020 PMC3971712

[10] Nottebohm F 1972 The origins of vocal learning. Am. Nat. 106, 116–140. (10.1086/282756)

[11] Ralls K, Fiorelli P, Gish S 1985 Vocalizations and vocal mimicry in captive harbor seals, *Phoca vitulina*. Can. J. Zool. 63, 1050–1056. (10.1139/z85-157)

[12] Stoeger AS, Mietchen D, Oh S, de Silva S, Herbst CT, Kwon S, Fitch WT 2012 An Asian elephant imitates human speech. Curr. Biol. 22, 2144–2148. (10.1016/j.cub.2012.09.022) 23122846 PMC3548412

[13] Richards DG, Wolz JP, Herman LM 1984 Vocal mimicry of computer-generated sounds and vocal labelling of objects by a bottlenosed dolphin, *Tursiops truncatus*. J. Comp. Psychol. 98, 10–28. (10.1037/0735-7036.98.1.10) 6705501

[14] Cranford TW, Amundin M, Norris KS 1996 Functional morphology and homology in the odontocete nasal complex: implications for sound generation. J. Morphol. 228, 223–285. (10.1002/(SICI)1097-4687(199606)228:3<223::AID-JMOR1>3.0.CO;2-3) 8622183

[15] Hayes KJ, Hayes C 1952 Imitation in a home-raised chimpanzee. J. Comp. Physiol. Psychol. 45, 450–459. (10.1037/h0053609) 13000013

[16] Winter P, Handley P, Ploog D, Schott D 1973 Ontogeny of squirrel monkey calls under normal conditions and under acoustic isolation. Behaviour 47, 230–239. (10.1163/156853973X00085) 4203637

[17] Hammerschmidt K, Jürgens U, Freudenstein T 2001 Vocal development in squirrel monkeys. Behaviour 138, 1179–1204. (10.1163/156853901753287190)

[18] Brockelman WY, Schilling D 1984 Inheritance of stereotyped gibbon calls. Nature 312, 634 (10.1038/312634a0) 6542175

[19] Owren MJ, Dieter JA, Seyfarth RM, Cheney DL 1992 ‘Food’ calls produced by adult female rhesus (*Macaca mulatta*) and Japanese (*M. fuscata*) macaques, their normally-raised offspring, and offspring cross-fostered between species. Behaviour 120, 218–231. (10.1163/156853992X00615)

[20] Jürgens U, Kirzinger A, von Cramon DY 1982 The effects of deep-reaching lesions in the cortical face area on phonation: a combined case report and experimental monkey study. Cortex 18, 125–139. (10.1016/S0010-9452(82)80024-5) 7187629

[21] Ploog D 1988 Neurobiology and pathology of subhuman vocal communication and human speech. In Primate vocal communication (eds Ploog D, Todt D, Goedeking P, Symmes D), pp. 195–212. Berlin, Germany: Springer.

[22] Jarvis ED 2007 Neural systems for vocal learning in birds and humans: a synopsis. J. Ornithol. 148, S35–S44. (10.1007/s10336-007-0243-0) PMC2726745 19684872

[23] Kroodsma DE, Konishi M 1991 A suboscine bird (Eastern Phoebe, *Sayornis phoebe*) develops normal song without auditory feedback. Anim. Behav. 42, 477–487. (10.1016/S0003-3472(05)80047-8)

[24] Scheiner E, Hammerschmidt K, Jürgens U, Zwirner P 2004 The influence of hearing impairment on preverbal emotional vocalizations of infants. Folia Phoniatr. Logop 56, 27–40. (10.1159/000075326) 14767158

[25] Marler P 2004 Bird calls: their potential for behavioral neurobiology. Ann. N Y Acad. Sci. 1016, 31–44. (10.1196/annals.1298.034) 15313768

[26] Myers RE 1976 Comparative neurology of vocalization and speech: proof of a dichotomy. Ann. N Y Acad. Sci. 280, 745–757. (10.1111/j.1749-6632.1976.tb25537.x) 827963

[27] Jarvis ED 2013 Evolution of brain pathways for vocal learning in birds and humans. In Birdsong, speech, and language: exploring the evolution of mind and brain, (eds Bolhuis JJ, Everaert M), pp. 63–107. Cambridge, MA: MIT Press.

[28] Simonyan K, Horwitz B 2011 Laryngeal motor cortex and control of speech in humans. Neuroscientist 17, 197–208. (10.1177/1073858410386727) 21362688 PMC3077440

[29] Kuypers HG 1958 Corticobulbar connexions to the pons and lower brain-stem in man: an anatomical study. Brain 81, 364–388. (10.1093/brain/81.3.364) 13596471

[30] Fitch WT, Huber L, Bugnyar T 2010 Social cognition and the evolution of language: constructing cognitive phylogenies. Neuron 65, 795–814. (10.1016/j.neuron.2010.03.011) 20346756 PMC4415479

[31] Doupe AJ, Kuhl PK 1999 Birdsong and human speech: common themes and mechanisms. Annu. Rev. Neurosci. 22, 567–631. (10.1146/annurev.neuro.22.1.567) 10202549

[32] Simpson HB, Vicario DS 1990 Brain pathways for learned and unlearned vocalizations differ in zebra finches. J. Neurosci. 10, 1541–1556. (10.1523/JNEUROSCI.10-05-01541.1990) 2332796 PMC6570078

[33] Petkov CI, Jarvis E 2012 Birds, primates, and spoken language origins: behavioral phenotypes and neurobiological substrates. Front. Evol. Neurosci. 16, 12 (10.3389/fnevo.2012.00012) PMC3419981 22912615

[34] Nowicki S 1989 Vocal plasticity in captive black-capped chickadees: the acoustic basis and rate of call convergence. Anim. Behav. 37, 64–73. (10.1016/0003-3472(89)90007-9)

[35] Hughes M, Nowicki S, Lohr B 1998 Call learning in black-capped chickadees (*Parus atricapillus*): the role of experience in the development of ‘chick-a-dee’ calls. Ethology 104, 232–249. (10.1111/j.1439-0310.1998.tb00065.x)

[36] Marler PR, Slabbekoorn H 2004 Nature's music: the science of birdsong. Amsterdam, The Netherlands: Elsevier.

[37] Snowdon CT, Elowson AM 1999 Pygmy marmosets modify call structure when paired. Ethology 105, 893–908. (10.1046/j.1439-0310.1999.00483.x)

[38] Weiss DJ, Garibaldi BT, Hauser MD 2001 The production and perception of long calls by cotton-top tamarins (*Saguinus oedipus*): acoustic analyses and playback experiments. J. Comp. Psychol. 115, 258–271. (10.1037/0735-7036.115.3.258) 11594495

[39] Egnor SER, Hauser MD 2004 A paradox in the evolution of primate vocal learning. Trends Neurosci. 27, 649–654. (10.1016/j.tins.2004.08.009) 15474164

[40] Mitani JC, Gros-Louis J 1998 Chorusing and call convergence in chimpanzees: tests of three hypotheses. Behaviour 135, 1041–1064. (10.1163/156853998792913483)

[41] Marshall AJ, Wrangham RW, Arcadi AC 1999 Does learning affect the structure of vocalizations in chimpanzees? Anim. Behav. 58, 825–830. (10.1006/anbe.1999.1219) 10512656

[42] Crockford C, Herbinger I, Vigilant L, Boesch C 2004 Wild chimpanzees produce group specific calls: a case for vocal learning? Ethology 110, 221–243. (10.1111/j.1439-0310.2004.00968.x)

[43] Watson SK, Townsend SW, Schel AM, Wilke C, Wallace EK, Cheng L, West V, Slocombe KE 2015 Vocal learning in the functionally referential food grunts of chimpanzees. Curr. Biol. 25, 495–499. (10.1016/j.cub.2014.12.032) 25660548

[44] Briefer EF, McElligott AG 2012 Social effects on vocal ontogeny in an ungulate, the goat, *Capra hircus*. Anim. Behav. 83, 991–1000. (10.1016/j.anbehav.2012.01.020)

[45] Lopez PT, Narins PM, Lewis ER, Moore SW 1988 Acoustically induced call modification in the white-lipped frog, *Leptodactylus albilabris*. Anim. Behav. 36, 1295–1308. (10.1016/s0003-3472(88)80198-2)

[46] Bolhuis JJ, Okanoya K, Scharff C 2010 Twitter evolution: converging mechanisms in birdsong and human speech. Nat. Rev. Neurosci. 11, 747 (10.1038/nrn2931) 20959859

[47] Tyack PL 2016 Vocal learning and auditory-vocal feedback. In Vertebrate sound production and acoustic communication (Springer Handbook of Auditory Research) (eds Suthers R, Fitch T, Popper AN, Fay RR), pp. 261–295. New York, NY: Springer.

[48] Tyack PL 2008 Convergence of calls as animals form social bonds, active compensation for noisy communication channels, and the evolution of vocal learning in mammals. J. Comp. Psychol. 122, 319–331. (10.1037/a0013087) 18729661

[49] Lombard E 1911 Le signe de l'elevation de la voix. Annales des Maladies de L'Oreille et du Larynx 37, 101–119.

[50] Brumm H, Zollinger SA 2011 The evolution of the Lombard effect: 100 years of psychoacoustic research. Behaviour 148, 1173–1198. (10.1163/000579511X605759)

[51] Parris KM, Velik-Lord M, North JM 2009 Frogs call at a higher pitch in traffic noise. Ecol. Soc. 14, 25 (10.5751/ES-02687-140125)

[52] Cunnington GM, Fahrig L 2010 Plasticity in the vocalizations of anurans in response to traffic noise. Acta Oecol. 36, 463–470. (10.1016/j.actao.2010.06.002)

[53] Lampe U, Reinhold K, Schmoll T 2014 How grasshoppers respond to road noise: developmental plasticity and population differentiation in acoustic signalling. Funct. Ecol. 28, 660–668. (10.1111/1365-2435.12215)

[54] Nonaka S, Takahashi R, Enomoto K, Katada A, Unno T 1997 Lombard reflex during PAG-induced vocalization in decerebrate cats. Neurosci. Res. 29, 283–289. (10.1016/S0168-0102(97)00097-7) 9527619

[55] Hage SR, Jürgens U, Ehret G 2006 Audio–vocal interaction in the pontine brainstem during self-initiated vocalization in the squirrel monkey. Eur. J. Neurosci. 3, 3297–3308. (10.1111/j.1460-9568.2006.04835.x) 16820019

[56] Eliades SJ, Wang X 2012 Neural correlates of the Lombard effect in primate auditory cortex. J. Neurosci. 32, 10 737–10 748. (10.1523/JNEUROSCI.3448-11.2012) PMC3752069 22855821

[57] Metzner W 1993 An audio-vocal interface in echolocating horseshoe bats. J. Neurosci. 13, 1899–1915. (10.1523/JNEUROSCI.13-05-01899.1993) 8478683 PMC6576583

[58] Elman JL 1981 Effects of frequency-shifted feedback on the pitch of vocal productions. J. Acoust. Soc. Am. 70, 45–50. (10.1121/1.386580) 7264071

[59] Fitch WT 2006 The biology and evolution of music: a comparative perspective. Cognition 100, 173–215. (10.1016/j.cognition.2005.11.009) 16412411

[60] Caldwell MC, Caldwell DK 1979 The whistle of the Atlantic bottlenosed dolphin *(Tursiops truncatus*)—ontogeny. In Behavior of marine animals, vol. 3, cetaceans (eds Winn HE and Olla BL), pp. 369–401. New York, NY: Plenum Press.

[61] Knörnschild M, Behr O, von Helversen O 2006 Babbling behavior in the sac-winged bat (*Saccopteryx bilineata*). Naturwissenschaften 93, 451–454. (10.1007/s00114-006-0127-9) 16736178

[62] Knörnschild M, Nagy M, Metz M, Mayer F, von Helversen O 2010 Complex vocal imitation during ontogeny in a bat. Biol. Lett. 6, 156–159. (10.1098/rsbl.2009.0685) 19812069 PMC2865031

[63] Marler P, Nelson DA 1993 Action-based learning: a new form of developmental plasticity in bird song. Neth. J. Zool. 43, 91–103. (10.1163/156854293X00232)

[64] Takahashi DY, Fenley AR, Teramoto Y, Narayanan DZ, Borjon JI, Holmes P, Ghazanfar AA 2015 The developmental dynamics of marmoset monkey vocal production. Science 349, 734–738. (10.1126/science.aab1058) 26273055

[65] Picardo MA et al. 2016 Population-level representation of a temporal sequence underlying song production in the zebra finch. Neuron 90, 866–876. (10.1016/j.neuron.2016.02.016) 27196976 PMC4941616

[66] Suge R, Okanoya K 2010 Perceptual chunking in the self-produced songs of Bengalese finches (*Lonchura striata* var. domestica). Anim. Cogn. 13, 515–523. (10.1007/s10071-009-0302-4) 20039089

[67] Pace F, Benard F, Glotin H, Adam O, White P 2010 Subunit definition and analysis for humpback whale call classification. Appl. Acoust. 71, 1107–1112. (10.1016/j.apacoust.2010.05.016)

[68] Strager H 1995 Pod-specific call repertoires and compound calls of killer whales, *Orcinus orca* Linnaeus, 1758, in the waters of northern Norway. Can. J. Zool. 73, 1037–1047. (10.1139/z95-124)

[69] Yurk H, Barrett-Lennard L, Ford JK, Matkin CO 2002 Cultural transmission within maternal lineages: vocal clans in resident killer whales in southern Alaska. Anim. Behav. 63, 1103–1119. (10.1006/anbe.2002.3012)

[70] Janik VM, Sayigh LS 2013 Communication in bottlenose dolphins: 50 years of signature whistle research. J. Comp. Physiol. A 199, 479–489. (10.1007/s00359-013-0817-7) 23649908

[71] Smotherman M, Knörnschild M, Smarsh G, Bohn K 2016 The origins and diversity of bat songs. J. Comp. Physiol. A 202, 535–554. (10.1007/s00359-016-1105-0) 27350360

[72] Holy TE, Guo Z 2005 Ultrasonic songs of male mice. PLoS Biol. 3, e386 (10.1371/journal.pbio.0030386) 16248680 PMC1275525

[73] Portfors CV, Perkel DJ 2014 The role of ultrasonic vocalizations in mouse communication. Curr. Opin Neurobiol. 28, 115–120. (10.1016/j.conb.2014.07.002) 25062471 PMC4177333

[74] Marler P 1997 Three models of song learning: evidence from behavior. J. Neurobiol. 33, 501–516. (10.1002/(SICI)1097-4695(19971105)33:5<501::AID-NEU2>3.0.CO;2-8) 9369456

[75] Warren DK, Patterson DK, Pepperberg IM 1996 Mechanisms of American English vowel production in a grey parrot (*Psittacus erithacus*). Auk 13, 41–58. (10.2307/4088934)

[76] Klatt DH, Stefanski RA 1974 How does a mynah bird imitate human speech? J. Acoust. Soc. Am. 55, 822–832. (10.1121/1.1914607) 4833078

[77] Fitch WT, de Boer B, Mathur N, Ghazanfar AA 2016 Monkey vocal tracts are speech-ready. Sci. Adv. 2, e1600723 (10.1126/sciadv.1600723) 27957536 PMC5148209

[78] Boë LJ, Berthommier F, Legou T, Captier G, Kemp C, Sawallis TR, Becker Y, Rey A, Fagot J 2017 Evidence of a vocalic proto-system in the baboon (*Papio papio*) suggests pre-hominin speech precursors. PLoS ONE 12, e0169321 (10.1371/journal.pone.0169321) 28076426 PMC5226677

[79] Stansbury AL, Janik VM 2019 Formant modification through vocal production learning in grey seals. Curr. Biol. 29, 1–6. (10.1016/j.cub.2019.05.071) 31231051

[80] Reichmuth C, Casey C 2014 Vocal learning in seals, sea lions, and walruses. Curr. Opin Neurobiol. 28, 66–71. (10.1016/j.conb.2014.06.011) 25042930

[81] Lilly JC 1965 Vocal mimicry in *Tursiops*: ability to match numbers and durations of human vocal bursts. Science 147, 300–301. (10.1126/science.147.3655.300) 17788214

[82] Eaton RL 1979 A beluga whale imitates human speech. Carnivore 2, 22–23.

[83] Ridgway S, Carder D, Jeffries M, Todd M 2012 Spontaneous human speech mimicry by a cetacean. Curr. Biol. 22, R860–R861. (10.1016/j.cub.2012.08.044) 23098588

[84] Murayama T, Iijima S, Katsumata H, Arai K 2014 Vocal imitation of human speech, synthetic sounds and beluga sounds, by a beluga (*Delphinapterus leucas*). Int. J. Comp. Psychol. 27, 369–384. (10.1007/978-1-4684-2985-5_9)

[85] Schevill WE, Lawrence B 1949 Underwater listening to the white porpoise (*Delphinapterus leucas*). Science 109, 143–144. (10.1126/science.109.2824.143) 17759169

[86] Janik VM 2014 Cetacean vocal learning and communication. Curr. Opin. Neurobiol. 28, 60–65. (10.1016/j.conb.2014.06.010) 25057816

[87] Favaro L, Neves S, Furlati S, Pessani D, Martin V, Janik VM 2016 Evidence suggests vocal production learning in a cross-fostered Risso's dolphin (*Grampus griseus*). Anim. Cogn. 19, 847–853. (10.1007/s10071-016-0961-x) 26874843

[88] Gannier A, Fuchs S, Quèbre P, Oswald JN 2010 Performance of a contour-based classification method for whistles of Mediterranean delphinids. Appl. Acoust. 71, 1063–1069. (10.1016/j.apacoust.2010.05.019)

[89] Abramson JZ, Hernández-Lloreda MV, García L, Colmenares F, Aboitiz F, Call J 2018 Imitation of novel conspecific and human speech sounds in the killer whale (*Orcinus orca*). Proc. R. Soc. B 285, 20172171 10.1098/rspb.2017.2171 PMC5805929 29386364

[90] Payne K, Payne R 1985 Large scale changes over 19 years in songs of humpback whales in Bermuda. Z. Tierpsychol. 68, 89–114. (10.1111/j.1439-0310.1985.tb00118.x)

[91] Guinee LN, Chu K, Dorsey EM 1983 Change over time in the songs of known individual humpback whales (*Megaptera novaeangliae*). In Communication and behavior of whales (ed. Payne R), pp. 59–80. Boulder, CO: Westview Press.

[92] Noad MJ, Cato DH, Bryden MM, Jenner MN, Jenner KC 2000 Cultural revolution in whale songs. Nature 408, 537 (10.1038/35046199) 11117730

[93] Stafford KM, Lydersen C, Wiig Ø, Kovacs KM 2018 Extreme diversity in the songs of Spitsbergen's bowhead whales. Biol. Lett. 14, 20180056 (10.1098/rsbl.2018.0056) 29618521 PMC5938564

[94] Vernes SC, Wilkinson GS 2019 Behaviour, biology and evolution of vocal learning in bats. Phil. Trans. R. Soc. B 375, 20190061 (10.1098/rstb.2019.0061) 31735153 PMC6895559

[95] Jones G, Ransome RD 1993 Echolocation calls of bats are influenced by maternal effects and change over a lifetime. Proc. R. Soc. Lond. B 252, 125–128. (10.1098/rspb.1993.0055) 8391702

[96] Boughman JW 1998 Vocal learning by greater spear-nosed bats. Proc. R. Soc. Lond. B 265, 227–233. (10.1098/rspb.1998.0286) PMC1688873 9493408

[97] Knörnschild M, Nagy M, Metz M, Mayer F, von Helversen O 2012 Learned vocal group signatures in the polygynous bat *Saccopteryx bilineata*. Anim. Behav. 84, 761–769. (10.1016/j.anbehav.2012.06.029)

[98] Prat Y, Azoulay L, Dor R, Yovel Y 2017 Crowd vocal learning induces vocal dialects in bats: playback of conspecifics shapes fundamental frequency usage by pups. PLoS Biol. 15, e2002556 (10.1371/journal.pbio.2002556) 29088225 PMC5663327

[99] Esser KH 1994 Audio-vocal learning in a non-human mammal: the lesser spear-nosed bat *Phyllostomus discolor*. Neuroreport 5, 1718–1720. (10.1097/00001756-199409080-00007) 7827315

[100] Prat Y, Taub M, Yovel Y 2015 Vocal learning in a social mammal: demonstrated by isolation and playback experiments in bats. Sci. Adv. 1, e1500019 (10.1126/sciadv.1500019) 26601149 PMC4643821

[101] Lattenkamp EZ, Vernes SC 2018 Vocal learning: a language-relevant trait in need of a broad cross-species approach. Curr. Opin. Behav. Sci. 21, 209–215. (10.1016/j.cobeha.2018.04.007)

[102] Jarvis ED 2006 Selection for and against vocal learning in birds and mammals. Ornithol. Sci. 5, 5–14. (10.2326/osj.5.5)

[103] Oelschläger HH, Ridgway SH, Knauth M 2010 Cetacean brain evolution: dwarf sperm whale (*Kogia sima*) and common dolphin (*Delphinus delphis*)—an investigation with high-resolution 3D MRI. Brain Behav. Evol. 75, 33–62. (10.1159/000293601) 20203478

[104] Manger PR, Pillay P, Maseko BC, Bhagwandin A, Gravett N, Moon DJ, Jillani N, Hemingway J 2009 Acquisition of brains from the African elephant (*Loxodonta africana*): perfusion-fixation and dissection. J. Neurosci. Methods 179, 16–21. (10.1016/j.jneumeth.2009.01.001) 19168095

[105] Ravignani A, Fitch W, Hanke FD, Heinrich T, Hurgitsch B, Kotz SA, Scharff C, Stoeger AS, de Boer B 2016 What pinnipeds have to say about human speech, music, and the evolution of rhythm. Front. Neurosci. 10, 274 (10.3389/fnins.2016.00274) 27378843 PMC4913109

